# Más allá del cambio metodológico en la práctica clínica: Evaluación de las pruebas de factor de crecimiento insulínico tipo 1

**DOI:** 10.1515/almed-2022-0092

**Published:** 2022-10-17

**Authors:** Paula Sienes Bailo, Marta Fabre Estremera, José Cuenca Alcocel, María Ángeles César Márquez

**Affiliations:** Department of Clinical Biochemistry, Miguel Servet University Hospital, Zaragoza, España; Aragon Institute of Health Research (IIS Aragon), Aragon, España

**Keywords:** comparación de métodos, factor de crecimiento insulínico tipo 1, hormona de crecimiento, inmunoensayo

## Abstract

**Objetivos:**

El factor de crecimiento insulínico tipo 1 (IGF-I) es el biomarcador más ampliamente utilizado para el diagnóstico y seguimiento de los trastornos relacionados con el crecimiento, aunque su cuantificación en suero implica una serie de dificultades. De este modo, se han observado discrepancias en las concentraciones de IGF-I según el tipo de ensayo empleado, especialmente en el contexto de concentraciones superiores o inferiores al rango de normalidad.

**Métodos:**

Entre noviembre y diciembre de 2020, se realizó un estudio prospectivo en un hospital universitario de tercer nivel, en el que se analizaron 212 muestras séricas para determinar la calidad analítica de la prueba de IGF-I cuando se realiza en el analizador Cobas e411 (Roche Diagnostics) y compararla con la de Immulite 2000XPi (Siemens).

**Resultados:**

El presente es el primer estudio en aportar evidencia sobre la existencia de discrepancias en las concentraciones de IGF-I, según sean medidas con Immulite 2000XPi o Cobas e411. En el análisis de regresión de Deming se obtuvo una pendiente de 1,570 (95% CI: 1,395–1,745), una ordenada en el origen de −58,591 (IC 95%: −89,151 to −28,030), con un R^2^=0,967 y un sesgo medio de +53,061, con una sobreestimación de los niveles de IGF-I. Observamos que con Cobas e411 se obtienen concentraciones de IGF-I anormalmente elevadas, aunque son necesarios más estudios para dilucidar la causa de dichas discrepancias.

**Conclusiones:**

Los resultados de este estudio pueden ser de utilidad para alertar a los facultativos, así como a los profesionales de laboratorio de dicha circunstancia, con el fin de evitar la interpretación errónea de niveles aumentados de IGF-I como un fracaso terapéutico en lugar de como un problema asociado a este cambio de método analítico.

El factor de crecimiento insulínico tipo 1(IGF-I) es una hormona polipeptídica de 70 aminoácidos que se sintetiza principalmente en el hígado, y que actúa como el principal mediador periférico de actividad de la hormona de crecimiento (GH). Esta hormona regula directamente la síntesis y secreción de IGF-I, siendo esta el principal marcador periférico de actividad de la GH y la que mejor correlación muestra con el estado de secreción de GH en la vida postnatal. Las fluctuaciones a corto plazo en la secreción de GH pueden ser causadas por multitud de factores, incluyendo la liberación pulsátil desde la glándula pituitaria, el ritmo circadiano y el sueño. Sin embargo, dichos factores tienen un efecto mínimo o ningún efecto en los niveles de IGF-I. Este hecho hace del IGF-I el biomarcador más ampliamente utilizado para el diagnóstico y seguimiento de los trastornos relacionados con el crecimiento, desde el déficit de GH hasta su exceso [1, 2].

Existen diversos tipos de inmunoensayos para la cuantificación del IGF-I en suero, que presentan problemas relacionados con su unión a las proteínas transportadoras, estabilidad a largo plazo de los patrones de calibración, reactividad cruzada con el IGF-II y desarrollo de intervalos de referencia (IR) fiables específicos según la edad y el sexo. Los ensayos suelen calibrarse empleando los patrones NIBSC 87/815 o NIBSC IS 02/254 de la Organización Mundial de la salud (OMS). No obstante, aunque se han realizado grandes avances en su armonización, aún se siguen observando discrepancias en las concentraciones de IGF-I dependiendo del ensayo empleado, especialmente si los resultados son superiores o inferiores al límite de normalidad. Esto podría deberse a diferencias en el diseño metodológico (competitivo, inmunométrico u otros), la especificad y afinidad de los anticuerpos, interferencias en la unión de proteínas, preparaciones de referencia y sensibilidad [3, 4].

De este modo, algunos autores proponen transformar las concentraciones de IGF-I en puntuaciones de desviación estándar (SDS) según la edad, sexo y etapa puberal, en caso de que se disponga de dichos valores, aunque la no normalidad de los valores de referencia de IGF-I complica enormemente el cálculo de este parámetro, obligando a recurrir al empleo de hojas de cálculo o a programas específicos para tal fin [2].

Por otro lado, la Federación Internacional de Química Clínica y Medicina de Laboratorio (IFCC) y el Instituto de Estándares Clínicos y de Laboratorio (CLSI) recomiendan a cada laboratorio que determinen sus propios IR [5]. El establecimiento de IR pediátricos es especialmente complicado, dados los problemas éticos adicionales asociados a la obtención de muestras de pacientes menores de edad. Como mínimo, cada laboratorio debería validar y verificar en su población los IR del fabricante o de los estudios publicados en la literatura. Es esencial verificar la similitud entre la población de referencia y la población atendida por el laboratorio, especialmente en aquellos parámetros que se ven influidos por la etnia, el sexo y la edad. Por lo tanto, los IR no deben aplicarse automáticamente en distintos entornos [6].

Los laboratorios clínicos conocen las diferentes estrategias a aplicar cuando se realiza un cambio de método. La veracidad de un método se puede evaluar aplicando las directrices EP-A2 o CLSI EP09 del CLSI, que indican cómo calcular el sesgo y la concordancia entre ensayos en los que se emplean muestras de pacientes. Se recomienda notificar cualquier cambio de método en el informe del laboratorio, con el fin de evitar la interpretación incorrecta de resultados obtenidos con otros métodos. Pueden existir diferencias significativas entre métodos, lo que puede derivar en cambios en el diagnóstico, inicio de tratamiento y gestión a largo plazo de los pacientes con trastornos relacionados con el crecimiento [3].

Describimos una situación ocurrida recientemente en nuestro laboratorio. Debido a un cambio en la licitación pública, se sustituyó la medición de IGF-I mediante ensayo inmunométrico enzimático por quimioluminiscencia (Immu-lite 2000XPi, Siemens Healthcare Diagnostics, UK) por un inmunoensayo de electroquimioluminiscencia (Cobas e411, Hoffmann-La Roche, Suiza). Previamente al cambio, se realizó un estudio de evaluación y se aceptó el cambio propuesto. En los informes de laboratorio se informaba sobre el empleo del nuevo método y se proporcionaban nuevos IR.

En unos pocos meses, se observó que un porcentaje sospechosamente elevado de las muestras presentaban concentraciones de IGF-I superiores a los IR específicos según el sexo, indicados por el nuevo fabricante. Así mismo, los facultativos también observaron discrepancias entre las mediciones de IGF-I realizadas con Cobas e411 y la evolución clínica de varios pacientes. Esto provocó un aumento en el número de consultas médicas y pruebas analíticas y de imagen destinadas a evaluar el estado de los pacientes.

Para comprobar si Cobas e411 estaba midiendo correctamente los niveles de IGF-I, se realizó un estudio prospectivo. En primer lugar, se comprobó dicho analizador y posteriormente se reevaluaron los resultados con las pruebas de IGF-I de Roche y Siemens. En el transcurso de un mes, se evaluaron muestras de suero seleccionadas aleatoriamente de pacientes habituales del laboratorio clínico del Hospital Universitario de Miguel Servet (Zaragoza, España). La gran mayoría de los pacientes procedían del Departamento de Pediatría y Endocrinología, a los que se sometía a seguimiento por patologías como acromegalia, retraso en el crecimiento o adenoma pituitario. En total, 114 de los 212 (53.8%) pacientes eran de sexo femenino, mientras que 98 (46.2%) eran de sexo masculino. El rango de edad fue de 2 a 90 años. Se recogieron las muestras en tubos de suero Vacutainer con gel separador (Becton, Dickinson and Company, NJ, EE.UU) y se centrifugaron tras la formación de coágulos. Las muestras se analizaron cada día con ambos ensayos. En ambos métodos (Cobas e411 and Immulite 200XPi), los calibradores empleados concordaban con el estándar de trazabilidad NIBSC IS 02/254 de la OMS. Cada día, previamente a la realización de los análisis, se procesaron los controles internos de la calidad de cada equipo a dos niveles de concentración, para garantizar la consistencia diaria del proceso analítico. Los objetivos de calidad fueron cumplir las especificaciones de la base de datos de variación biológica deseable (Tabla 1) [7]. El control se aceptaba si el resultado se encontraba en el 2° rango de la media indicada por el fabricante. Se extrajo información clínica detallada de la historia clínica electrónica de los pacientes. Para los datos de comparación entre métodos, se emplearon los análisis de regresión de Deming y Bland Altman. Los análisis estadísticos, cálculos y representaciones gráficas se realizaron con los programas de análisis estadístico y análisis de datos IBM SPSS Statistics 20.0 y XLSTAT 2021.4 (2021).

**Tabla 1:** Error total, sesgo e imprecisión de los dos niveles de control interno de la calidad de los ensayos.

	Calidad deseable de la base de datos de variación biológica	Cobas e411		Immulite 2000XPi	
		IQC1	IQC2	IQC1	IQC2
% CV	4,70	4,07	3,59	2,33	2,65
% Sesgo	7,15	5,30	4,81	4,70	1,69
% ET	14,9	12,02	10,73	8,55	6,07

ET, error total; CV, coeficiente de variación; IQC, control interno de la calidad.

Las gráficas de regresión de Deming y de Bland Altman se muestran en la Figura 1. Los resultados de IGF-I obtenidos con el analizador Cobas e411 e Immulite 2000XPi mostraron una buena correlación (R^2^=0,967) con una pendiente de 1,570 (intervalo de confianza del 95% (IC): 1,395–1,745) y una intersección de −58,591 (IC 95%: −89,151 to −28,030) (Figura 1A). Los resultados de IGF-I obtenidos con Cobas e411 solían ser superiores a los de Immulite 2000XPi. Obtuvimos un sesgo medio de 53,061 unidades. En las concentraciones más elevadas, el sesgo entre datos era aún mayor (Figura 1B). Como ejemplo ilustrativo de dichas discrepancias, presentamos el caso de una paciente de 12 años con déficit de la hormona de crecimiento en tratamiento con rhGH durante los últimos cinco años (Caso 27). Entre marzo de 2017 y septiembre de 2019, los niveles de IGF-I de esta paciente se midieron con Immulite 2000XPi. A partir de octubre de 2019, se empezó a emplear el analizador Cobas e411. A partir de entonces, los niveles de IGF-I superaban el IR y no reflejaban su estado clínico. En noviembre de 2020, se midieron las concentraciones de IGF-I con los dos ensayos. En la Figura 2 se muestran las concentraciones de IGF-I de la paciente.

**Figura 1:**
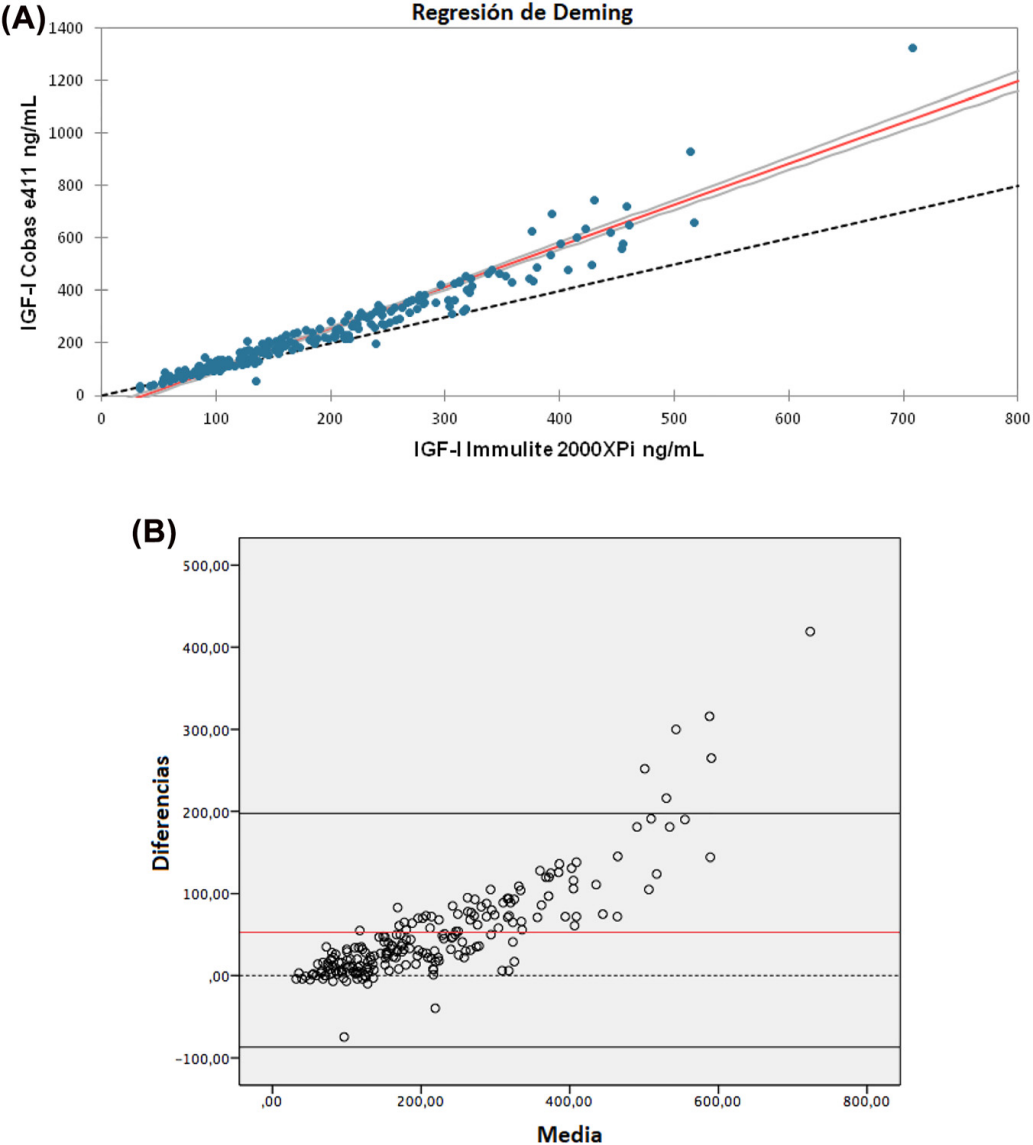
Datos de comparación de métodos. (A) Regresión de Deming. Gráfico de dispersión de valores de IGF-1 medidos con cada plataforma. La línea roja representa la línea de regresión. La línea discontinua representa los valores de IGF-1. (B) Gráfica de Bland-Alman. Las líneas horizontales negras representan la diferencia media entre los valores de Inmulite y Roche, y los límites superior e inferior del intervalo de confianza del 95%. Los puntos representan los valores IGF-1 obtenidos con cada método. La línea roja representa la diferencia media.

**Figura 2:**
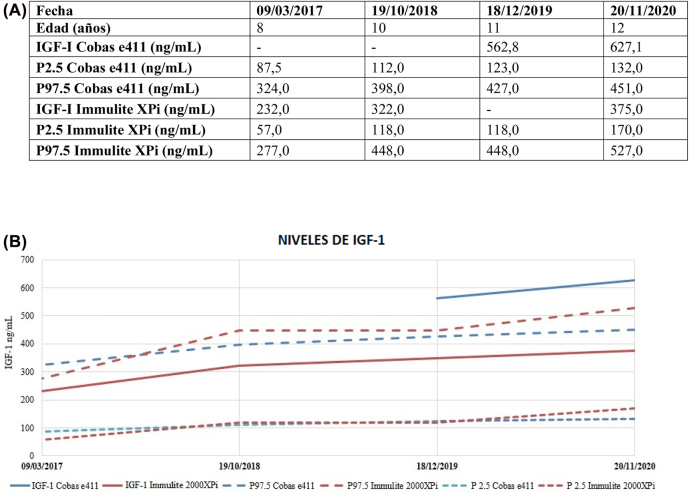
Niveles de IGF-I Los percentiles P2.5 y P97.5 corresponden a los límites inferior y superior de los intervalos de referencia de cada técnica. (A) Tabla de los niveles de IGF-I en los últimos 4 años. (B) Evolución de los niveles de IGF-I en los últimos 4 años.

Los niveles medios de IGF-I circulante fueron aumentando con relativa lentitud durante la infancia, para aumentar gradualmente durante la etapa prepuberal y puberal, alcanzando su nivel máximo en la etapa IV-V de Tanner, a partir de la cual fueron disminuyendo [8]. La hormona IGF-I alcanza sus niveles máximos en suero a los 15 años de edad [9]. De este modo, aunque se pueden encontrar concentraciones elevadas en este grupo etario, no es lógico encontrar tantos valores superiores al IR en pacientes sin síntomas clínicos. Además, los niveles anormalmente elevados de IGF-I obtenidos con Cobas e411 no se justifican por una falta de correlación entre equipos (R^2^=0,967) o por interferencias endógenas comunes como hemólisis, lipemia, ictericia o biotina. Tampoco parece deberse a factores exógenos como el tratamiento con rhGH. Los estudios realizados por el fabricante descartan esta posible interferencia. Así mismo, también hemos hallado en nuestra serie pacientes sin tratamiento que muestran importantes diferencias entre ensayos.

Se está realizando un número creciente de estudios para evaluar y comparar métodos de medición de analitos comunes, como la hemoglobina A1c, marcadores cardíacos y hepáticos [10], [11], [12]. No obstante, la evidencia sobre analitos más específicos como los marcadores hormonales aún es muy limitada. Teóricamente, el uso de diferentes métodos analíticos no debería representar un problema en la práctica clínica, ya que en los kits comerciales con los que se obtienen valores superiores, se deberían establecer también límites de normalidad superiores, con el fin de lograr una correcta clasificación de los pacientes. Sin embargo, multitud de laboratorios aún se encuentran ante este problema, por lo que es necesario tener en cuenta los hallazgos de este estudio y comprobar que aquellos métodos con los que se obtienen valores superiores de algunos analitos también tienen límites de normalidad superiores que se correlacionen con dichos valores. En el presente estudio, demostramos que el 35.5% de los resultados de IGF-I medido con Cobas e411 excedieron el IR, frente al 16.5% de los medidos con Immulite 2000XPi (Datos Suplementarios, Tabla S1). Este fenómeno ha derivado en un incremento en el volumen de consultas y peticiones de análisis en nuestro hospital. Tras este estudio, y de acuerdo con el Departamento de Pediatría, se decidió volver a emplear el analizador Immulite 2000XPi.

Este estudio presenta algunas limitaciones. En primer lugar, se adoptaron los IR de los fabricantes. En segundo lugar, nuestro grupo de trabajo no puedo dilucidar la causa de las discrepancias entre los niveles de IGF-I medidos con Cobas e411 y el estado clínico del paciente. Es necesario realizar más estudios para hallar la causa de dichas discrepancias. Además, el índice kappa habría añadido valor al estudio, pero no disponíamos de los datos necesarios para calcularlo. No obstante, el propósito de la presente comunicación es alertar a otros profesionales sobre esta circunstancia.

El presente estudio es el primero en aportar evidencia de que en el analizador Cobas e411 se obtienen niveles de IGF-I superiores a los obtenidos con Immulite 2000XPi. Los facultativos podrían interpretar dichos niveles superiores de IGF-I como un fracaso terapéutico, aunque podría tratarse de una interpretación incorrecta debido al método analítico empleado. Así mismo, los estudios de intercomparabilidad llevados a cabo mostraron una buena qcorrelación entre los dos métodos. No obstante, esto no garantiza la ausencia de problemas a la hora de cambiar de método para medir algunos analitos, especialmente en el caso del IGF-I, cuando se emplea para el seguimiento de los tratamientos, debiendo establecerse IR específicos según la edad y el sexo.

## Supplementary Material

Supplementary Material

## Material Suplementario

La versión en línea de este artículo incluye material suplementario (https://doi.org/10.1515/almed-2022-0092).
